# Lanostane-Type Triterpenoids from *Scilla scilloides* and Structure Revision of Drimiopsin D

**DOI:** 10.1007/s13659-015-0076-0

**Published:** 2015-10-12

**Authors:** Fu-Cai Ren, Li-Xia Wang, Qin Yu, Xian-Jun Jiang, Fei Wang

**Affiliations:** BioBioPha Co., Ltd., Kunming, 650201 People’s Republic of China

**Keywords:** *Scilla scilloides*, Lanostane, Scillascillol, Scillascillone, Structure revision

## Abstract

**Abstract:**

Two hitherto unknown lanostane-type triterpenoids, namely scillascillol (**1**) and scillascillone (**2**), and a hitherto unknown norlanostane-triterpene glycoside, namely scillascilloside B-1 (**3**), were isolated from the ethanol extract of the whole plants of *Scilla scilloides*. Their structures were elucidated on the basis of extensive spectroscopic studies. In addition, the structure of drimiopsin D (**6a**) has been revised as 2,5-dimethoxy-8-methyl-1,3,6-trihydroxyxanthone (**6**) by reanalysis of the spectroscopic data.

**Graphical Abstract:**

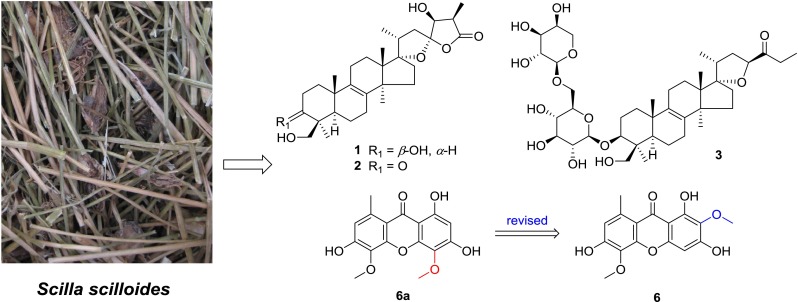

**Electronic supplementary material:**

The online version of this article (doi:10.1007/s13659-015-0076-0) contains supplementary material, which is available to authorized users.

## Introduction

**Fig. 1:**
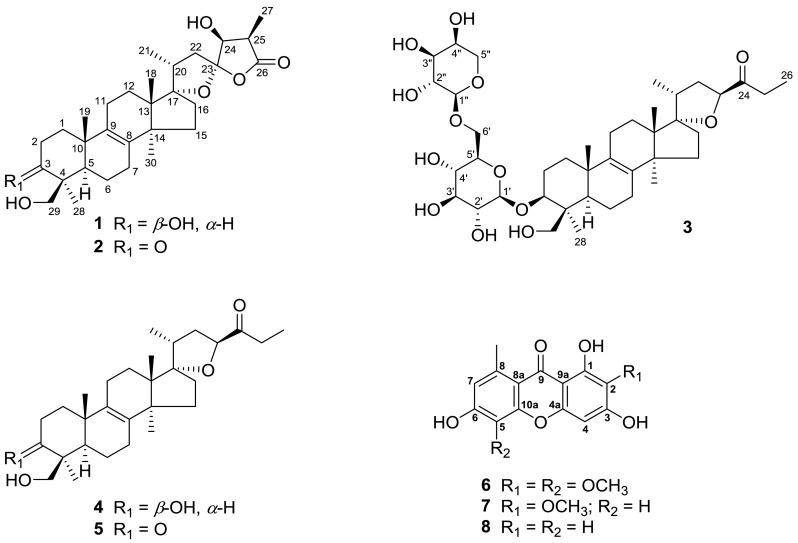
Structures of compounds **1–8**

*Scilla scilloides* (Lindl.) Druce is a perennial herb belonging to the Liliaceae family, also compiled in Hyacinthaceae family in the relatively new classification system [1]. The bulbs or the whole plants have been used as a foodstuff, a traditional Chinese medicine for promoting blood circulation, an analgesic, an anti-inflammatory agent, and treatment of heart failure and arrhythmia [2]. Previous chemical investigations on *S. scilloides* reported homoisoflavones, lanostane-type and norlanostane-type triterpenoids, xanthones, lignans, etc. [3–10]. As part of a BioBioPha (http://www.chemlib.cn) objective to assemble a large-scale natural product library valuable in the discovery of new drug leads from nature [11–14], the phytochemical investigation on the whole plants of *S. scilloides* led to isolation of two new lanostane-type triterpenoids, namely scillascillol (**1**) and scillascillone (**2**), a new norlanostane-triterpene glycoside, namely scillascilloside B-1 (**3**), together with two known norlanostanes, 15-deoxoeucosterol (**4**) [4] and 3-dehydro-15-deoxoeucosterol (**5**) [4], and three known xanthones, drimiopsin D (**6**) [15], drimiopsin C (**7**) [15] and norlichexanthone (**8**) [16] (Fig. 1). This paper describes the isolation and structural elucidation of new lanostane-type triterpenoids and structure revision of a xanthone drimiopsin D.

## Results and Discussion

**Fig. 2:**
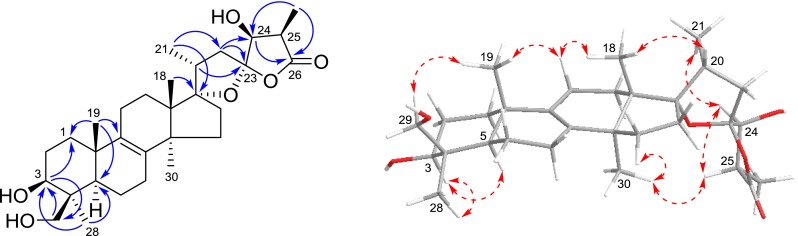
Key HMBC () and ROESY () correlations of **1**

Compound **1** was obtained as a white amorphous powder. Its molecular formula was determined to be C_30_H_46_O_6_ on the basis of negative-ion HRESIMS at *m/z* 501.3207 [M − H]^−^ (calcd for C_30_H_45_O_6_, 501.3216). The IR absorption bands at 3441, 3432 and 1765 cm^−1^ suggested the presence of hydroxy and carbonyl functionalities, respectively. The ^1^H NMR spectrum (Table 1) of **1** indicated signals due to four tertiary methyls [*δ*_H_ 0.89 (s), 1.01 (s), 1.24 (s), 1.52 (s)], two secondary methyls [*δ*_H_ 1.07 (d, *J* = 6.8 Hz); 1.47 (d, *J* = 7.1 Hz)], a pair of oxygenated methylene protons [*δ*_H_ 3.70, 4.56 (each d, *J* = 11.0 Hz)], and two oxygenated methine protons [*δ*_H_ 3.61 (dd, *J* = 11.6, 4.4 Hz); 4.34 (d, *J* = 4.4 Hz)]. The ^13^C NMR and DEPT spectrum (Table 1) exhibited 30 carbon signals, including an ester carbonyl resonance at [*δ*_C_ 178.9 (s)], a tetra-substituted double bond at [*δ*_C_ 135.1 (s), 134.9 (s)], and five oxygenated carbons at [*δ*_C_ 64.5 (t), 77.4 (d), 80.0 (d), 99.2 (s), 117.0 (s)]. By analyzing the above signals, especially the set of particular quaternary carbons [*δ*_C_ 99.2, 117.0, 178.9] and an oxygenated methine [*δ*_H_ 4.34 (d, *J* = 4.4 Hz); *δ*_C_ 77.4 (d)], compound **1** was presumed as a kind of characteristic lanostane triterpenoid derived from this genus. The above NMR spectroscopic features of **1** were very similar to those of scillasaponin D [17] and scillasaponin G [18], except lack of the signals due to the sugar moiety and an up-field shift about 10 ppm of the ^13^C NMR signal at C-3 of **1**. Based on analysis of the above spectral data, a planar structure of **1** was assumed, which was also confirmed by the HMBC (Fig. 2) correlations from H-3 to C-1 and C-29, from Me-19 to C-1 and C-9, as well as from Me-28 and H-29 to C-3. Taking into account its biological source and characteristic NMR data (including chemical shifts and coupling constants) of the spiro rings, the stereochemistry of **1** should be consistent with those analogues reported from this genus [8]. The hydroxy group at C-3 was equatorial (*β*) according to an axial–axial coupling between H-3 and H-2*β* (*J* = 11.6 Hz) and an axial-equatorial coupling between H-3 and H-2*α* (*J* = 4.4 Hz), which was also supported by the ROESY (Fig. 2) correlations of H-3 ↔ H-5 and H-3 ↔ Me-28. Considering a relatively good rigid property of spiro rings, the determination of its stereochemistry was able to be conducted on the basis of ROESY analysis. The ROESY correlations of H-24 ↔ Me-21, H-25 ↔ Me-30 and Me-30 ↔ H-15*α* were indicative of *α*-orientation of these protons, while the correlations of Me-18 ↔ H-20/H-11*β* indicative of their *β*-orientation. Therefore, the structure of **1** was determined as 17*α*,23*α*-epoxy-3*β*,24*β*,29-trihydroxylanost-8-en-26,23-olide and named scillascillol.

**Table 1:** NMR spectroscopic data of scillascillol (**1**) and scillascillone (**2)** in pyridine-*d*_5_ (*δ*_H_ 8.71, *δ*_C_ 149.9 ppm)

No.	**1**		**2**	
	*δ*_H_	*δ*_C_	*δ*_H_	*δ*_C_
1	1.19 (td, 13.3, 3,4, H-α)	35.8 t	1.48 (td, 13.1, 4.4, H-α)	37.1 t
	1.69 (dt, 13.3, 3.4, H-β)		1.90 (ddd, 13.1, 5.6, 2.9, H-β)	
2	1.95 (m)	29.0 t	2.37 (ddd, 14.2, 4.4, 2.9, H-α)	36.3 t
	2.03 (m)		2.86 (td, 14.2, 5.7, H-β)	
3	3.61 (dd, 11.6, 4.4)	80.0 d		214.5 s
4		43.2 s		55.0 s
5	1.26 (dd, 12.8, 1.6)	51.5 d	1.69 (m)	53.2 d
6	1.49 (m)	18.9 t	1.69 (m)	19.7 t
	1.81 (m)		1.77 (m)	
7	1.99 (m)	26.8 t	2.01 (m)	26.5 t
8		135.1 s		135.9 s
9		134.9 s		134.2 s
10		37.2 s		37.2 s
11	1.99 (m)	21.0 t	2.01 (m)	21.1 t
	2.13 (m)		2.05 (m)	
12	1.42 (m)	24.9 t	1.44 (m)	24.9 t
	2.25 (dt, 13.0, 9.0)		2.25 (dt, 13.2, 8.7)	
13		48.8 s		48.7 s
14		50.7 s		50.7 s
15	1.32 (br. t, 10.6)	31.9 t	1.31 (br. t, 10.2)	31.8 t
	1.65 (br. d, 9.1)		1.64 (m)	
16	1.82 (m)	37.8 t	1.82 (ddd, 14.7, 11.1, 1.5)	37.7 t
	2.65 (dt, 14.5, 8.9)		2.65 (dt, 14.7, 8.9)	
17		99.2 s		99.1 s
18	0.89 (s)	18.7 q	0.90 (s)	18.7 q
19	1.01 (s)	20.1 q	1.27 (s)	19.6 q
20	2.18 (m)	43.9 d	2.18 (m)	43.9 d
21	1.07 (d, 6.8)	18.7 q	1.06 (d, 6.8)	18.7 q
22	2.52 (dd, 14.1, 6.5)	38.5 t	2.52 (dd, 13.9, 6.5)	38.4 t
	2.69 (d, 14.1)		2.70 (d, 13.9)	
23		117.0 s		117.0 s
24	4.34 (d, 4.4)	77.4 d	4.35 (dd, 4.7, 3.8)^a^	77.4 d
25	3.27 (m)	41.3 d	3.28 (m)	41.3 d
26		178.9 s		178.8 s
27	1.47 (d, 7.1)	9.0 q	1.47 (d, 7.1)	9.0 q
28	1.52 (s)	23.4 q	1.47 (s)	20.7 q
29	3.70 (d, 11.0)	64.5 t	3.85 (d, 11.2)	65.1 t
	4.56 (d, 11.0)		4.42 (d, 11.2)	
30	1.24 (s)	26.0 q	1.23 (s)	25.9 q

^a^Coupling constants derived from 24-OH [7.89 (d, 4.7)] and H-25

Compound **2** was isolated as a white amorphous powder. The HRESIMS (neg.) at *m/z* 499.3053 [M − H]^−^ (calcd for C_30_H_43_O_6_, 499.3060) indicated the molecular formula C_30_H_44_O_6_, corresponding to nine degrees of unsaturation. By comparison with the reported NMR data [17, 18], the signals of characteristic five-membered spiro rings, a tetra-substituted double bond and six methyls were detected as before, which indicated that **2** had a very similar structure to scillascillol (**1**). The main difference is only that **2** had a newly arising keto carbon [*δ*_C_ 214.5 (s)] instead of the oxygenated methine signal [*δ*_H_ 3.61 (dd, *J* = 11.6, 4.4 Hz); *δ*_C_ 80.0 (d)] at C-3 of **1**. Thus, compound **2** should be the 3-dehydro derivative of **1**. And this was confirmed by the HMBC correlations from H-1 to C-3, C-5, C-9 and C-10, as well as from Me-28 and H-29 to C-3, C-4 and C-5. Therefore, the structure of **2** was determined as 24*β*,29-dihydroxy-17*α*,23*α*-epoxy-3-oxolanost-8-en-26,23-olide and named scillascillone.

**Fig. 3:**
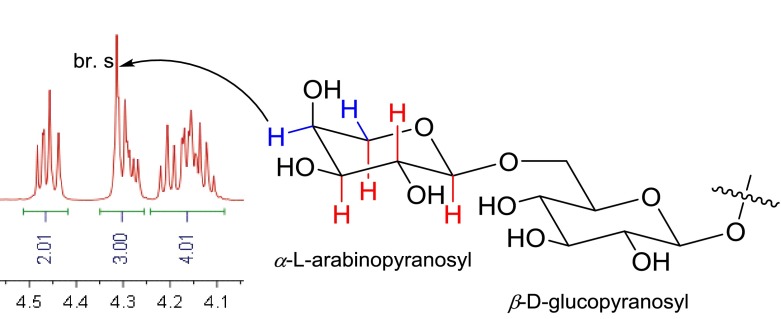
Chair conformation of sugar moiety in **3**

Compound **3** was also obtained as a white amorphous powder, and its molecular formula was deduced to be C_40_H_64_O_13_ based on its negative-ion HRESIMS at *m/z* 751.4261 [M − H]^−^ (calcd for C_40_H_63_O_13_, 751.4269). The ^1^H NMR spectrum (Table 2) of **3** was characterized by signals due to four tertiary methyls [*δ*_H_ 1.48 (s), 1.47 (s), 0.90 (s), 0.87 (s)], one secondary methyl [*δ*_H_ 1.00 (d, *J* = 6.6 Hz)], one primary methyl [*δ*_H_ 1.05 (t, *J* = 7.3 Hz)], and two anomeric protons [*δ*_H_ 4.98 (d, *J* = 7.9 Hz); 4.92 (d, *J* = 7.0 Hz)]. The ^13^C NMR spectrum (Table 2) of **3** implied the presence of a tetra-substituted double bond [*δ*_C_ 135.2 (s), 134.6 (s)], one keto carbonyl [*δ*_C_ 212.6 (s)], and two anomeric carbons [*δ*_C_ 106.2 (d), 105.6 (d)]. On the other hand, a set of additional 11 oxygenated carbons were detected by comparison with those of 15-deoxoeucosterol (**4**), suggesting the presence of a hexose and a pentose. The assignment of the hexose as C-6 glycosylated glucopyranosyl was launched by observation of a set of specific oxygenated carbons at [*δ*_C_ 106.2 (d), 75.4 (d), 78.7 (d), 72.0 (d), 77.1 (d), 69.9 (t)] and a diagnostic glycosidation shift (∆ ≈ +8 ppm) of C-6 of glucopyranosyl. It was still supported by the detailed HMBC analysis and comparison with the reported spectral data [19]. The remaining carbons at [*δ*_C_ 105.6 (d), 72.3 (d), 74.4 (d), 69.3 (d), 66.7 (t)] were established as a terminal *α*-l-arabinopyranosyl unit by analysis of coupling constants of sugar moiety (Fig. 3), in which axial–axial couplings between H-1 and H-2 (*J* = 7.0 Hz), and between H-2 and H-3 (*J* = 8.5 Hz), and axial-equatorial coupling between H-3 and H-4 (*J* = 2.8 Hz) were clearly observed. And the above-mentioned NMR feature was also consistent with the reported spectral data [20, 21]. In addition, on acid hydrolysis, **3** afforded monosaccharides in the aqueous layer, which were found to be identical with authentic samples of l-arabinose and d-glucose by chiral HPLC analysis. The connection of these units was established by the HMBC correlations from the anomeric proton of the arabinopyranosyl at [*δ*_H_ 4.92 (d, *J* = 7.0 Hz)] to C-6 of the glucopyranosyl at [*δ*_C_ 69.9 (t)], and from the anomeric proton of the glucopyranosyl at [*δ*_H_ 4.98 (d, *J* = 7.9 Hz)] to C-3 of the aglycone at [*δ*_C_ 88.9 (d)]. Thus, compound **3** was concluded to be 15-deoxoeucosterol 3-*O*-*α*-l-arabinopyranosyl-(1 → 6)-*β*-d-glucopyranoside, and named scillascilloside B-1.

**Table 2:** NMR spectroscopic data of scillascilloside B-1 (**3)** in pyridine-*d*_5_ (*δ*_H_ 8.71, *δ*_C_ 149.9 ppm)

No.	*δ*_H_	*δ*_C_	No.	*δ*_H_	*δ*_C_
1	1.12 (m)	35.7 t	20	2.02 (m)	43.7 d
	1.64 (m)		21	1.00 (d, 6.6)	17.3 q
2	2.03 (m)	27.6 t	22	1.75 (m)	36.9 t
	2.39 (m)			1.98 (m)	
3	3.50 (dd, 11.7, 4.6)	88.9 d	23	4.63 (dd, 10.4, 7.4)	81.7 s
4		44.4 s	24		212.6 s
5	1.11 (br. d, 11.7, 4.6)	51.7 d	25	2.54 (m)	32.4 t
6	1.42 (m)	18.7 t	26	1.05 (t, 7.3)	7.8 q
	1.75 (m)		28	1.47 (s)	23.1 q
7	1.99 (m)	26.9 t	29	3.63 (d, 11.1)	63.2 t
8		135.2 s		4.45 (d, 11.1)	
9		134.6 s	30	1.48 (s)	26.5 q
10		36.7 s	1′	4.98 (d, 7.9)	106.2 d
11	1.90 (m)	21.1 t	2′	3.95 (dd, 7.9, 8.6)	75.4 d
	2.06 (m)		3′	4.21 (t, 8.6)	78.7 d
12	1.40 (m)	25.3 t	4′	4.12 (t, 8.6)	72.0 d
	2.34 (m)		5′	4.15 (m)	77.1 d
13		48.9 s	6′	4.28 (m)	69.9 t
14		50.8 s		4.92 (m)	
15	1.38 (m)	32.1 t	1″	4.92 (d, 7.0)	105.6 d
	1.63 (m)		2″	4.47 (dd, 8.5, 7.0)	72.3 d
16	1.59 (m	39.8 t	3″	4.17 (dd, 8.5, 2.8)	74.4 d
	2.16 (m)		4″	4.31 (br. s)	69.3 d
17		97.1 s	5″	3.74 (br. d, 10.5)	66.7 t
18	0.87 (s)	19.3 q		4.30 (br. d, 10.5)	
19	0.90 (s)	19.6 q

**Fig. 4:**
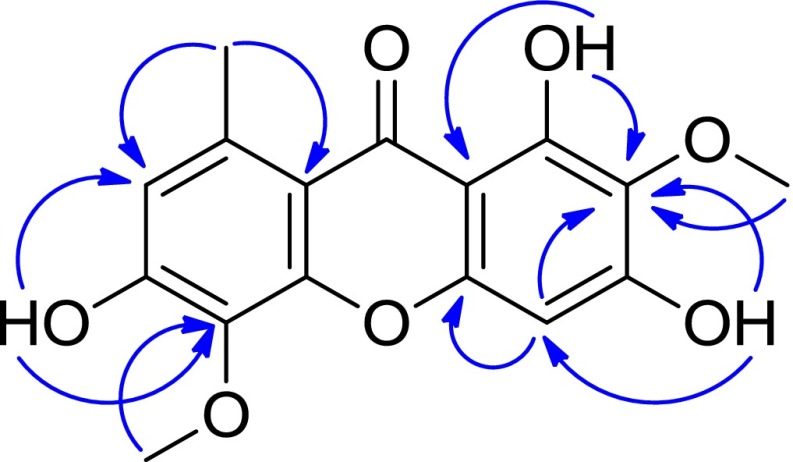
Key HMBC () correlations of **6**

Compound **6**, a yellow amorphous powder, had a molecular formula of C_16_H_14_O_7_ based on HRESIMS (neg.) at *m/z* 317.0665 [M − H]^−^ (calcd for C_16_H_13_O_7_, 317.0661). The ^1^H NMR spectrum (Table 3) indicated the presence of two methoxy signals [*δ*_H_ 3.72 (s), 3.81 (s)], one aromatic methyl [*δ*_H_ 2.66 (s)], two aromatic singlets [*δ*_H_ 6.41, 6.69], and three low-field exchangeable proton singlets at [*δ*_H_ 10.73, 10.79, 13.44]. The ^13^C NMR spectrum (Table 3) displayed a total of 16 carbon resonances ascribable to two benzene rings, two methoxy group [*δ*_C_ 60.0 (q), 60.8 (q)], one methyl [*δ*_C_ 22.8 (q)], and one carbonyl carbon [*δ*_C_ 182.1 (s)]. The above NMR spectroscopic features were very similar to those of drimiopsin I [22], and the most significant difference was from an additional methoxy signal in **6**. The methoxy group was positioned at C-2 according to the HMBC correlations (Fig. 4) from 1-OH at [*δ*_H_ 13.44 (s)] and 2-OCH_3_ at [*δ*_H_ 3.72 (s)] to C-2 at [*δ*_C_ 130.6 (s)]. All of these data for **6** were consistent with the structure 2,5-dimethoxy-8-methyl-1,3,6-trihydroxyxanthone.

**Table 3:** NMR spectroscopic data of drimiopsin D (**6)**

No.	**6** ^a^		**6** ^b^	
	*δ*_H_	*δ*_C_	*δ*_H_	*δ*_C_
1		154.6 s		155.9 s
2		130.6 s		131.9 s
3		158.0 s		158.9 s
4	6.41 (s)	93.5 d	6.37 (s)	94.4 d
4a		151.7 s		153.3 s
5		132.8 s		134.2 s
6		155.2 s		156.4 s
7	6.69 (s)	116.0 d	6.61 (s)	116.8 d
8		136.2 s		138.5 s
8a		111.1 s		113.0 s
9		182.1 s		183.9 s
9a		102.3 s		104.1 s
10a		151.7 s		153.7 s
8-CH_3_	2.66 (s)	22.8 q	2.70 (s)	23.3 q
2-OCH_3_	3.72 (s)	60.0 q	3.85 (s)	60.9 q
5-OCH_3_	3.81 (s)	60.8 q	3.90 (s)	61.7 q
1-OH	13.44 (s)			
3-OH	10.79 (br. s)			
6-OH	10.73 (br. s)			

^a^Measured in DMSO-*d*_6_ (*δ*_H_ 2.49, *δ*_C_ 39.5 ppm)

^b^Measured in CD_3_OD (*δ*_H_ 3.30, *δ*_C_ 49.0 ppm)

**Fig. 5:**
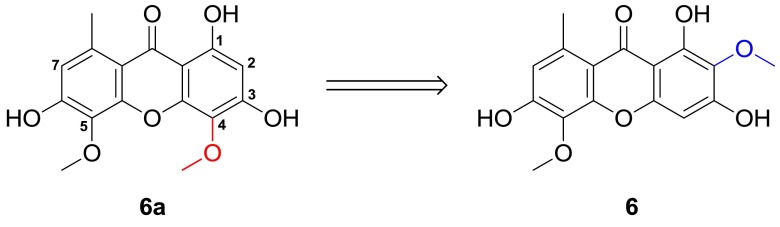
Structure revision of drimiopsin D

It was worth mentioning that the ^1^H NMR data of **6** were identical to drimiopsin D (**6a**) isolated by D.A. Mulholland from *Drimiopsis maculata* [15]. However, there were some deviation in aspect of the ^13^C NMR data of **6** and **6a**. We believed that the ^13^C NMR data of **6a** should be measured in CD_3_OD. This inference was subsequently confirmed, in view of the complete consistency of the spectral data of **6** and **6a**, when we re-tested its ^13^C NMR using CD_3_OD as deuterated solvent. As a conclusion, the structure of drimiopsin D (**6a**) was revised as 2,5-dimethoxy-8-methyl-1,3,6-trihydroxyxanthone (**6**) (Fig. 5).

## Experimental

### General Experimental Procedures

Optical rotations were measured on Jasco P-1020 or SGW_®_-3 (INESA Instrument Co., Ltd., Shanghai, China) automatic digital polarimeter. UV data were obtained from HPLC online analysis. IR spectra (KBr) were obtained on a Bruker Tensor-27 infrared spectrophotometer. NMR spectra were carried out on a Bruker Avance III 600 or Bruker AV-400 (Bruker BioSpin GmbH, Rheinstetten, Germany) spectrometer with deuterated solvent signals used as internal standards. ESIMS and HRESIMS were measured using Bruker HCT Esquire 3000 and API QSTAR time-of-flight mass spectrometers, respectively. Silica gel (200–300 mesh, Qingdao Marine Chemical Inc., China), MCI gel CHP-20P (75–150 μm, Mitsubishi Chemical Corporation, Japan), Chromatorex C-18 (40–75 μm, Fuji Silysia Chemical Ltd., Japan) and Sephadex LH-20 (Amersham Biosciences, Sweden) were used for column chromatography. Fractions were monitored and analyzed using TLC, in combination with Agilent 1200 series HPLC system equipped by Extend-C18 column (5 μm, 4.6 × 150 mm). A TCI Chiral MB-S column (5 μm, 4.6 × 250 mm, Tokyo Chemical Industry Co., Ltd., Tokyo, Japan) was applied for determination of acid hydrolyzates using Agilent 1200 series HPLC system with an external Alltech 3300 ELSD detector (Grace, Deerfield, USA).

### Plant Material

The whole plants of *S. scilloides* were collected from He County of Anhui Province, China, in August 2009, and identified by Prof. Shou-Jin Liu of Anhui University of Chinese Medicine. A voucher specimen (No. BBP0208018SS) was deposited at BioBioPha Co., Ltd.

### Extraction and Isolation

The dried and crushed whole plants of *S. scilloides* (9.5 kg) were extracted with EtOH-H_2_O (95:5, v/v; 3 × 18 L, each 7 days) at room temperature, and the solvent was removed under reduced pressure to give crude extract (ca. 1.9 kg), which was fractionated by silica gel CC successively eluted with a gradient of increasing acetone in petroleum ether (PE) (15:1, 10:1, 7:1, 4:1, 2:1, 1:1, 0:1; v/v) and then MeOH to afford fractions A–H, respectively. Fraction B was isolated by Sephadex LH-20 CC (CHCl_3_/MeOH; 1:1) and repeated silica gel CC (CHCl_3_/MeOH; 100:0 → 100:1) to yield **5** (181 mg). Fraction C was further separated by silica gel CC (CHCl_3_/MeOH; 100:1 → 20:1), Sephadex LH-20 (MeOH) and preparative TLC (PE/EtOAc; 8:1) to provide **2** (49 mg), **4** (474 mg) and **8** (45 mg). Fraction D was applied repeatedly to RP-18 CC (MeOH/H_2_O; 60 → 90 %) and then by Sephadex LH-20 (MeOH) to afford **1** (40 mg), **6** (127 mg) and **7** (116 mg). Compound **3** (291 mg) was eventually acquired by means of repeated silica gel CC (CHCl_3_/MeOH; 30:1 → 10:1), RP-18 (MeOH/H_2_O; 40 → 70 %) and Sephadex LH-20 (CHCl_3_/MeOH; 1:1) from the fraction F.

### Scillascillol (**1**)

White amorphous powder; [α]_D_^25^ −0.7 [*c* 0.42, MeOH-CHCl_3_ (1:1)]; IR (KBr) ν_max_: 3441, 3432, 2943, 2880, 1765, 1631, 1455, 1374, 1321, 1116, 1036, 997, 944, 923 cm^−1^; ^1^H and ^13^C NMR data: see Table 1; ESIMS (pos.): *m/z* 525 [M + Na]^+^; HRESIMS (neg.): *m/z* 501.3207 [M − H]^−^ (calcd for C_30_H_45_O_6_, 501.3216).

### Scillascillone (**2**)

White amorphous powder; [α]_D_^25^ +46.0 (*c* 0.25, MeOH); IR (KBr) ν_max_: 3471, 2960, 2943, 1765, 1702, 1639, 1456, 1381, 1320, 1115, 1046, 998, 943, 923 cm^−1^; ^1^H and ^13^C NMR data: see Table 1; ESIMS (pos.): *m/z* 523 [M + Na]^+^; HRESIMS (neg.): *m/z* 499.3053 [M − H]^−^ (calcd for C_30_H_43_O_6_, 499.3060).

### Scillascilloside B-1 (**3**)

White amorphous powder; [α]_D_^25^ −57.9 (*c* 0.30, MeOH); IR (KBr) ν_max_: 3440, 2940, 2883, 1774, 1725, 1713, 1633, 1456, 1376, 1255, 1078, 1048, 1010, 755 cm^−1^; ^1^H and ^13^C NMR data: see Table 2; ESIMS (pos.): *m/z* 775 [M + Na]^+^; HRESIMS (neg.): *m/z* 751.4261 [M − H]^−^ (calcd for C_40_H_63_O_13_, 751.4269).

### 2,5-Dimethoxy-8-methyl-1,3,6-trihydroxyxanthone (= drimiopsin D, **6**)

Yellow amorphous powder; UV (MeOH) λ_max_: 247, 276 (sh), 321 nm; IR (KBr) ν_max_: 3443, 2959, 2849, 1650, 1619, 1579, 1513, 1461, 1320, 1292, 1267, 1186, 1162, 1105, 992, 816, 796 cm^−1^; ^1^H and ^13^C NMR data: see Table 3; ESIMS (pos.): *m/z* 341 [M + Na]^+^; HRESIMS (neg.): *m/z* 317.0665 [M − H]^−^ (calcd for C_16_H_13_O_7_, 317.0661).

### Acid Hydrolysis of **3**

Compound **3** (3 mg) was heated in 2 M HCl (3 mL) at a temperature of 45 °C for 4 h. After cooling, the reaction mixture was neutralized with KOH to a pH of approximately 7, and then extracted with EtOAc. The aqueous layer was analyzed by HPLC under the following conditions: solvent, n-hexane/isopropanol (85:15); flow rate, 1.0 ml/min; temperature, 25 °C. The retention times (*t*_R_) of d-glucose and l-arabinose were 5.5 and 6.9 min, respectively.

## Electronic supplementary material

Supplementary material 1 (DOCX 2005 kb)
