# Supplementary material for: Lanostane-Type Triterpenoids from Scilla scilloides and Structure Revision of Drimiopsin D
Source: Nat Prod Bioprospect. 2015 Oct 12;5(5):263–70. doi: 10.1007/s13659-015-0076-0 (PMC4607680; doi:10.1007/s13659-015-0076-0)

**Electronic Supplementary Material**

**Lanostane-type triterpenoids from *Scilla scilloides* and structure revision of drimiopsin D**

Fu-Cai Ren, Li-Xia Wang, Qin Yu, Xian-Jun Jiang, Fei Wang*

BioBioPha Co., Ltd., Kunming 650201, People’s Republic of China

*To whom correspondence should be addressed.

E-mail: f.wang@mail.biobiopha.com

**Content list:**

**S1.** ^1^H NMR spectrum (600 MHz, Pyridine-*d*_5_) of scillascillol (**1**).

**S2.** ^13^C NMR spectrum (150 MHz, Pyridine-*d*_5_) of scillascillol (**1**).

**S3.** HSQC spectrum (600 MHz, Pyridine-*d*_5_) of scillascillol (**1**).

**S4.** HMBC spectrum (600 MHz, Pyridine-*d*_5_) of scillascillol (**1**).

**S5.** ROESY spectrum (600 MHz, Pyridine-*d*_5_) of scillascillol (**1**).

**S6.** ^1^H NMR spectrum (600 MHz, Pyridine-*d*_5_) of scillascillone (**2**).

**S7.** ^13^C NMR spectrum (150 MHz, Pyridine-*d*_5_) of scillascillone (**2**).

**S8.** HSQC spectrum (600 MHz, Pyridine-*d*_5_) of scillascillone (**2**).

**S9.** HMBC spectrum (600 MHz, Pyridine-*d*_5_) of scillascillone (**2**).

**S10.** ROESY spectrum (600 MHz, Pyridine-*d*_5_) of scillascillone (**2**).

**S11.** ^1^H NMR spectrum (600 MHz, Pyridine-*d*_5_) of scillascilloside B-1 (**3**).

**S12.** ^13^C NMR spectrum (150 MHz, Pyridine-*d*_5_) of scillascilloside B-1 (**3**).

**S13.** HSQC spectrum (600 MHz, Pyridine-*d*_5_) of scillascilloside B-1 (**3**).

**S14.** HMBC spectrum (600 MHz, Pyridine-*d*_5_) of scillascilloside B-1 (**3**).

**S15.** ROESY spectrum (600 MHz, Pyridine-*d*_5_) of scillascilloside B-1 (**3**).

**S16.** ^1^H NMR spectrum (400 MHz, DMSO-*d*_6_) of drimiopsin D (**6**).

**S17.** ^13^C NMR spectrum (100 MHz, DMSO-*d*_6_) of drimiopsin D (**6**).

**S18.** HMBC spectrum (600 MHz, DMSO-*d*_6_) of drimiopsin D (**6**).

**S19.** ^1^H NMR spectrum (600 MHz, CD_3_OD) of drimiopsin D (**6**).

**S20.** ^13^C NMR spectrum (100 MHz, CD_3_OD) of drimiopsin D (**6**).

**S21.** HMBC spectrum (600 MHz, CD_3_OD) of drimiopsin D (**6**).

**S1.** ^1^H NMR spectrum (600 MHz, Pyridine-*d*_5_) of scillascillol (**1**).


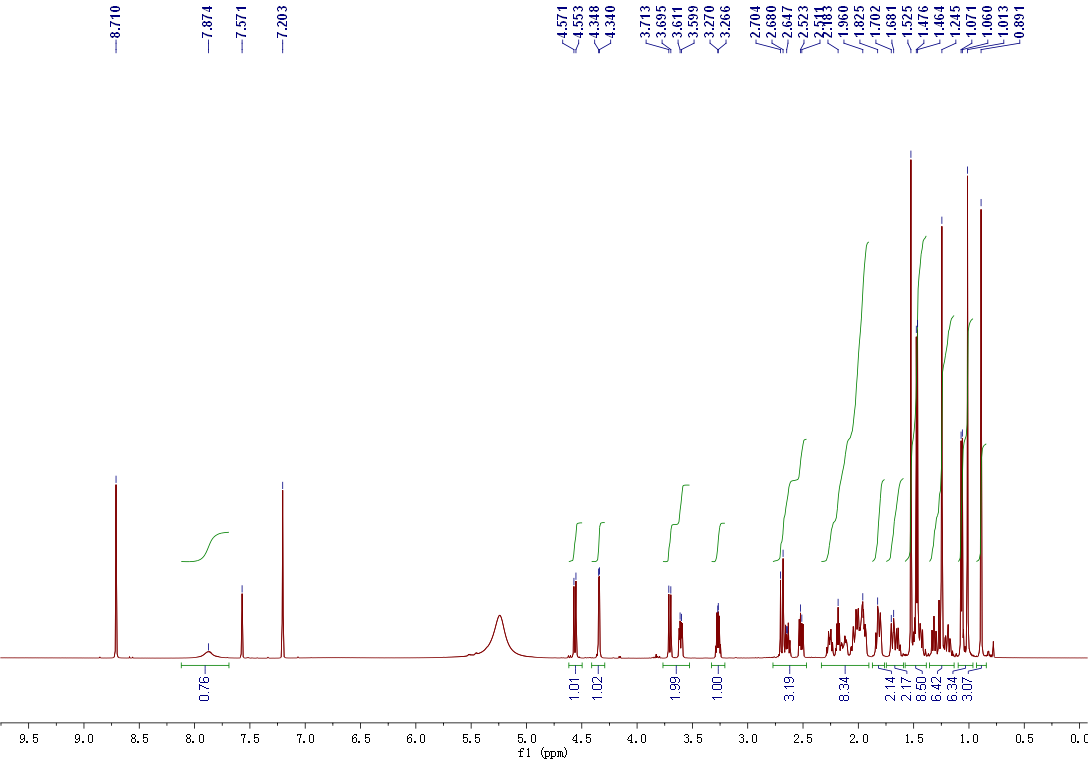

**S2.** ^13^C NMR spectrum (150 MHz, DMSO-*d*_6_) of scillascillol (**1**).


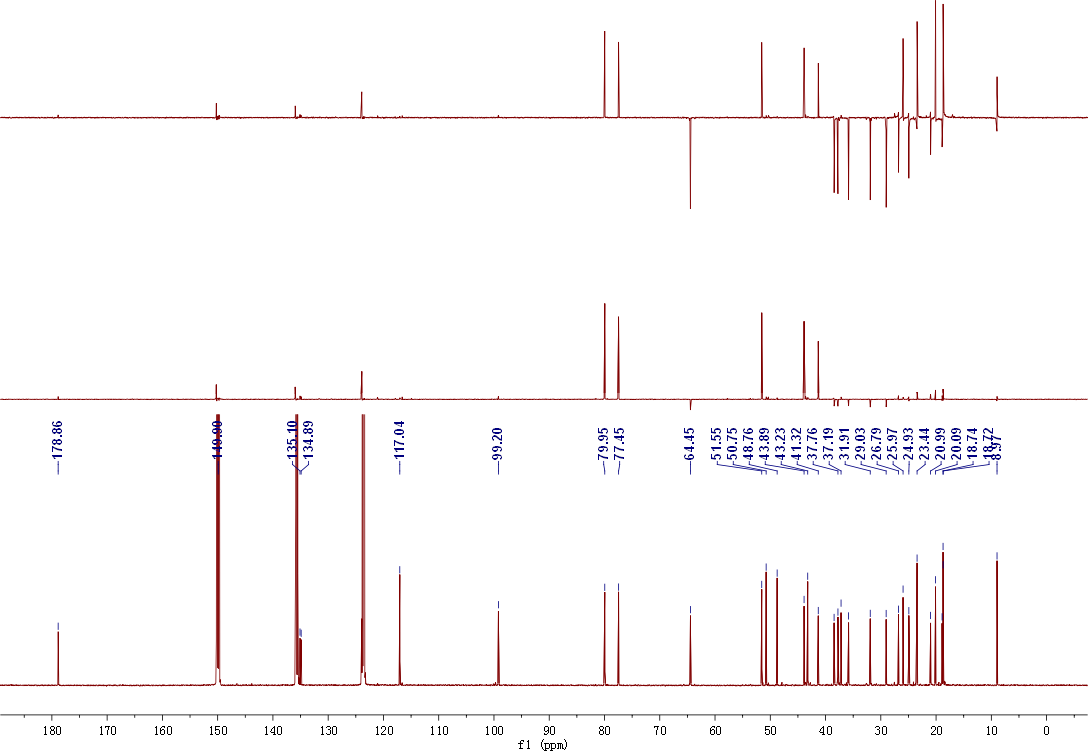

**S3.** HSQC spectrum (600 MHz, Pyridine-*d*_5_) of scillascillol (**1**).


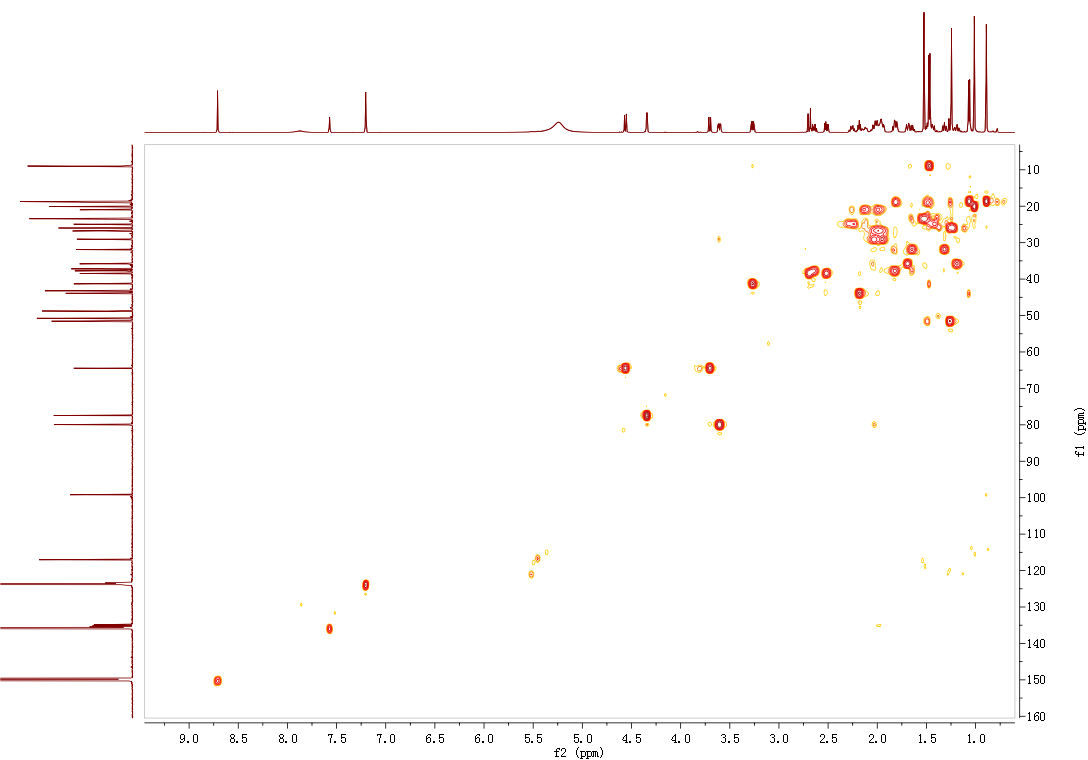

**S4.** HMBC spectrum (600 MHz, Pyridine-*d*_5_) of scillascillol (**1**).


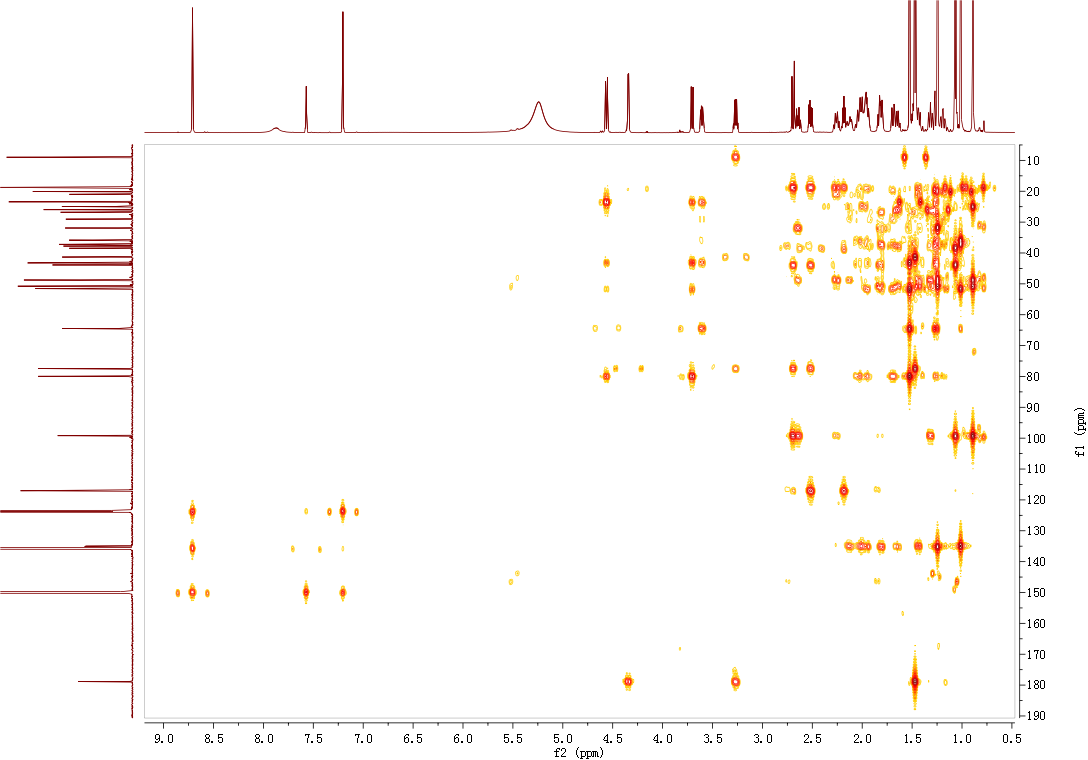

**S5.** ROESY spectrum (600 MHz, Pyridine-*d*_5_) of scillascillol (**1**).


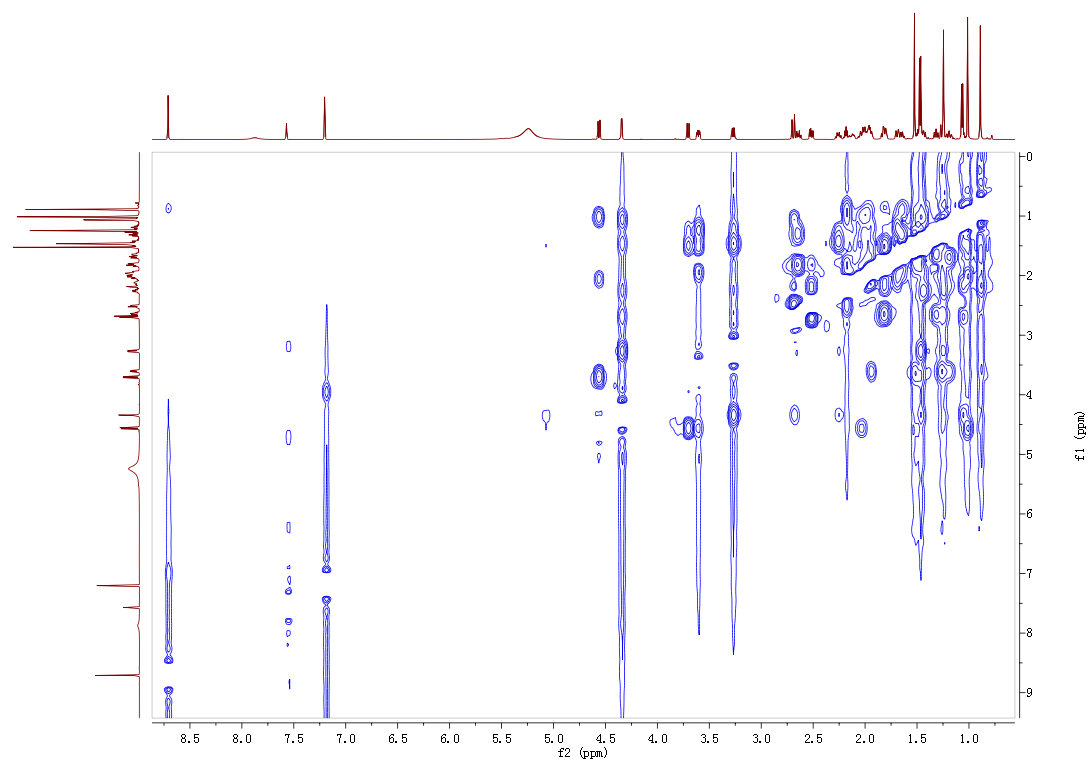

**S6.** ^1^H NMR spectrum (600 MHz, Pyridine-*d*_5_) of scillascillone (**2**).


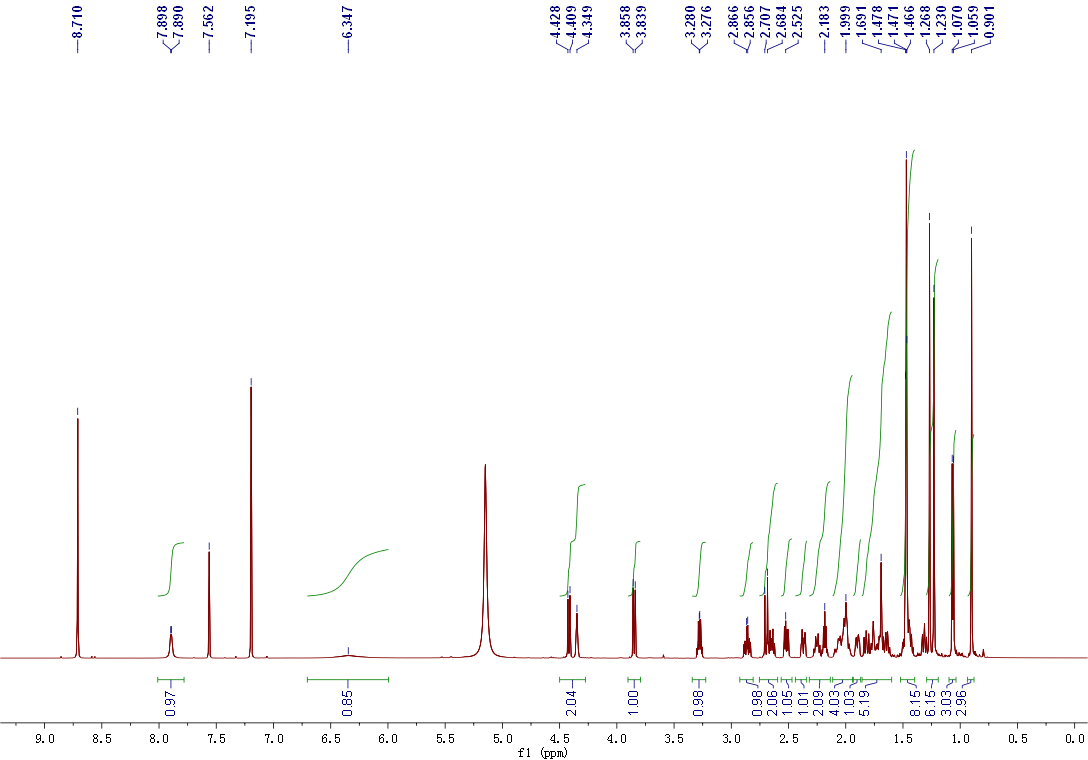

**S7.** ^13^C NMR spectrum (150 MHz, Pyridine-*d*_5_) of scillascillone (**2**).


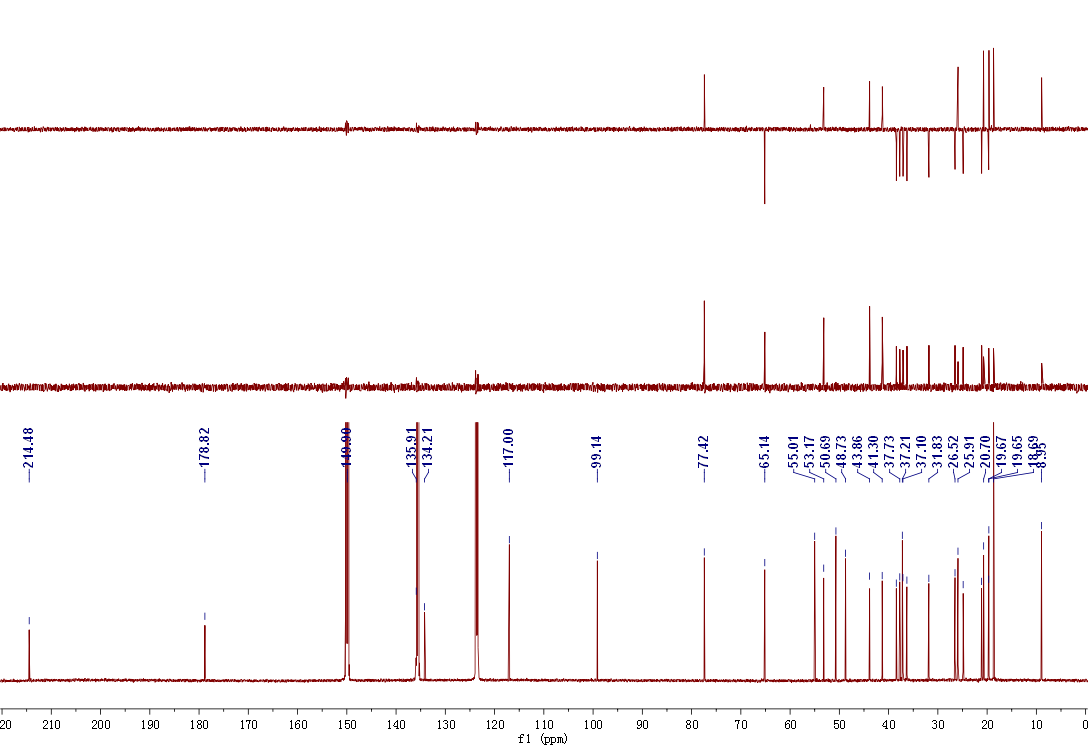

**S8.** HSQC spectrum (600 MHz, Pyridine-*d*_5_) of scillascillone (**2**).


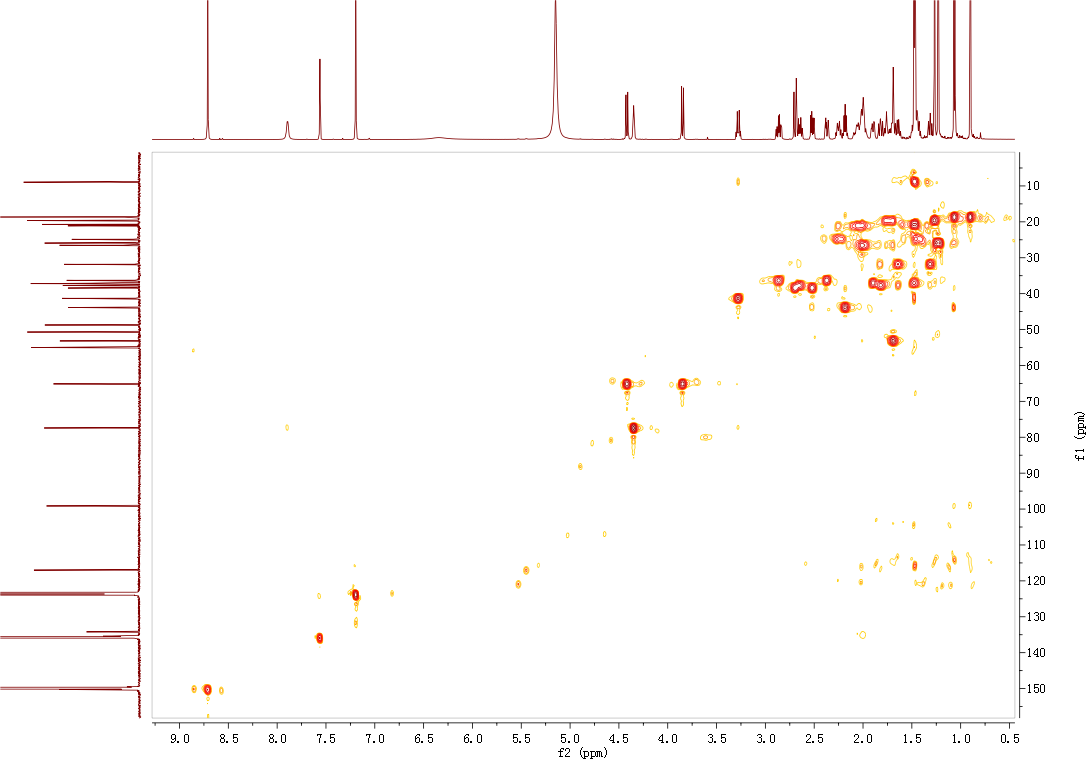

**S9.** HMBC spectrum (600 MHz, Pyridine-*d*_5_) of scillascillone (**2**).


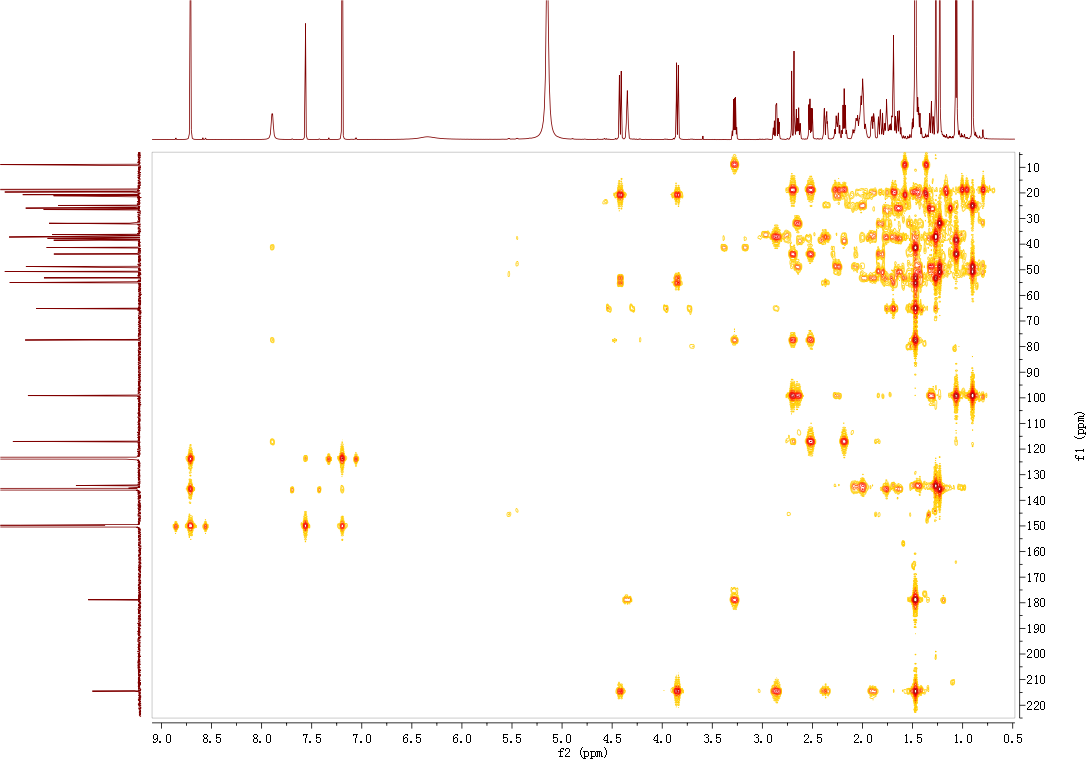

**S10.** ROESY spectrum (600 MHz, Pyridine-*d*_5_) of scillascillone (**2**).


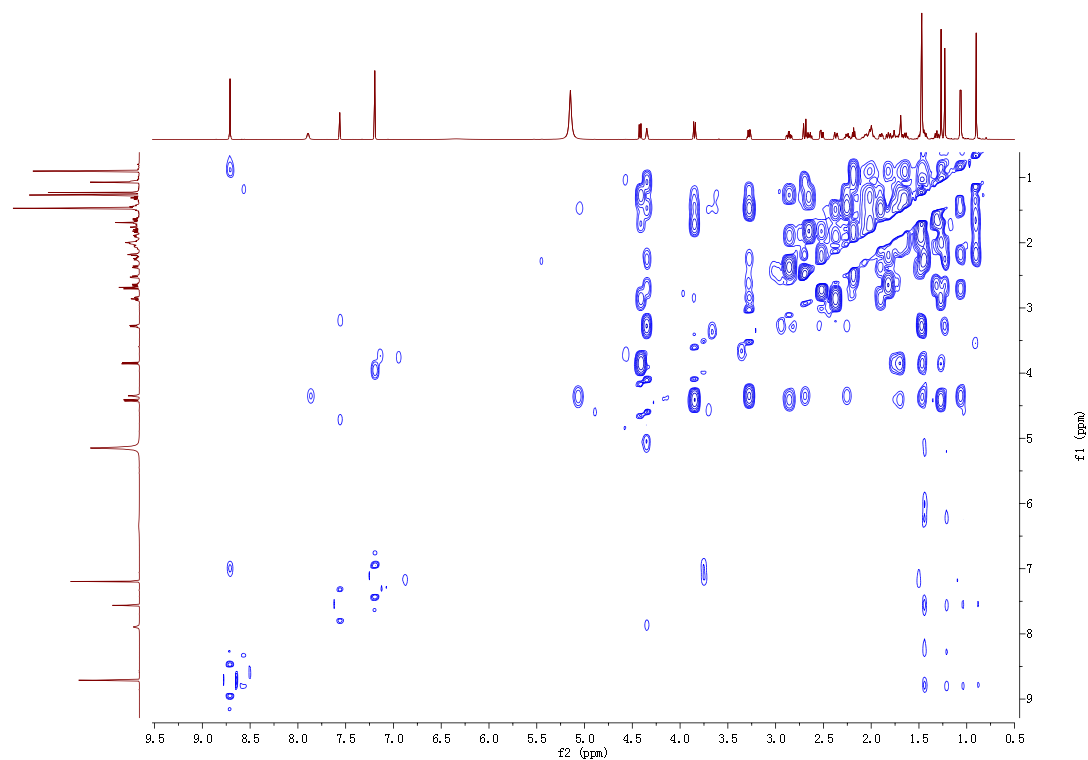

**S11.** ^1^H NMR spectrum (600 MHz, Pyridine-*d*_5_) of scillascilloside B-1 (**3**).


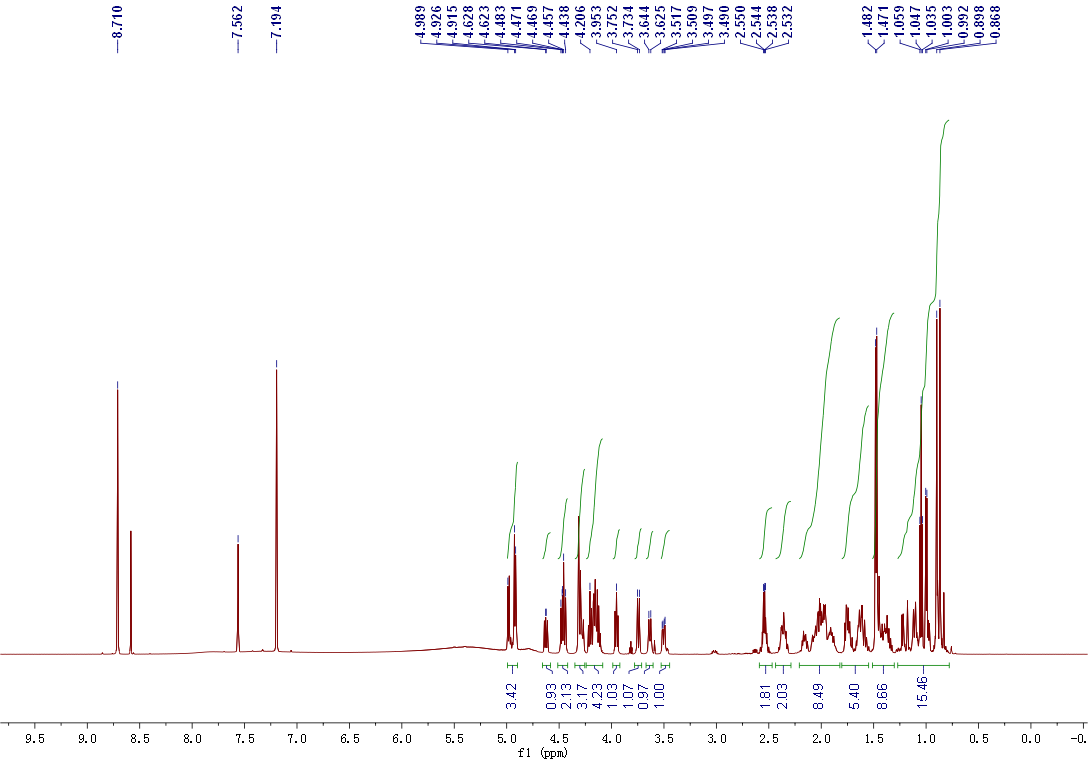

**S12.** ^13^C NMR spectrum (150 MHz, Pyridine-*d*_5_) of scillascilloside B-1 (**3**).


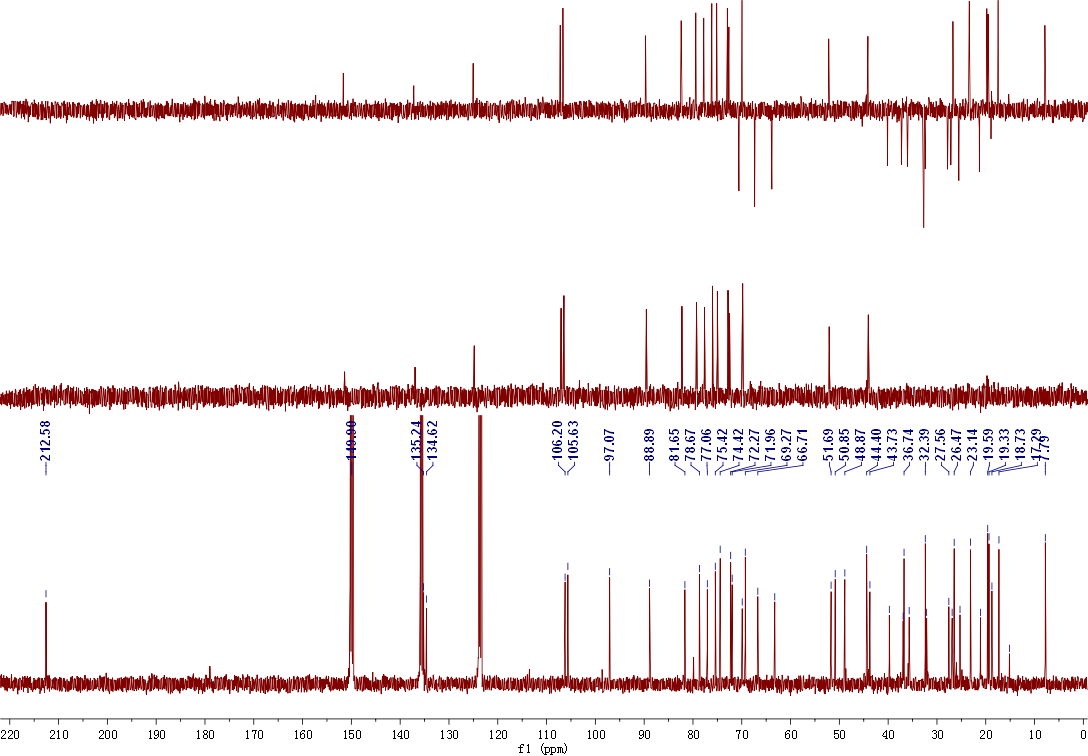

**S13.** HSQC spectrum (600 MHz, Pyridine-*d*_5_) of scillascilloside B-1 (**3**).


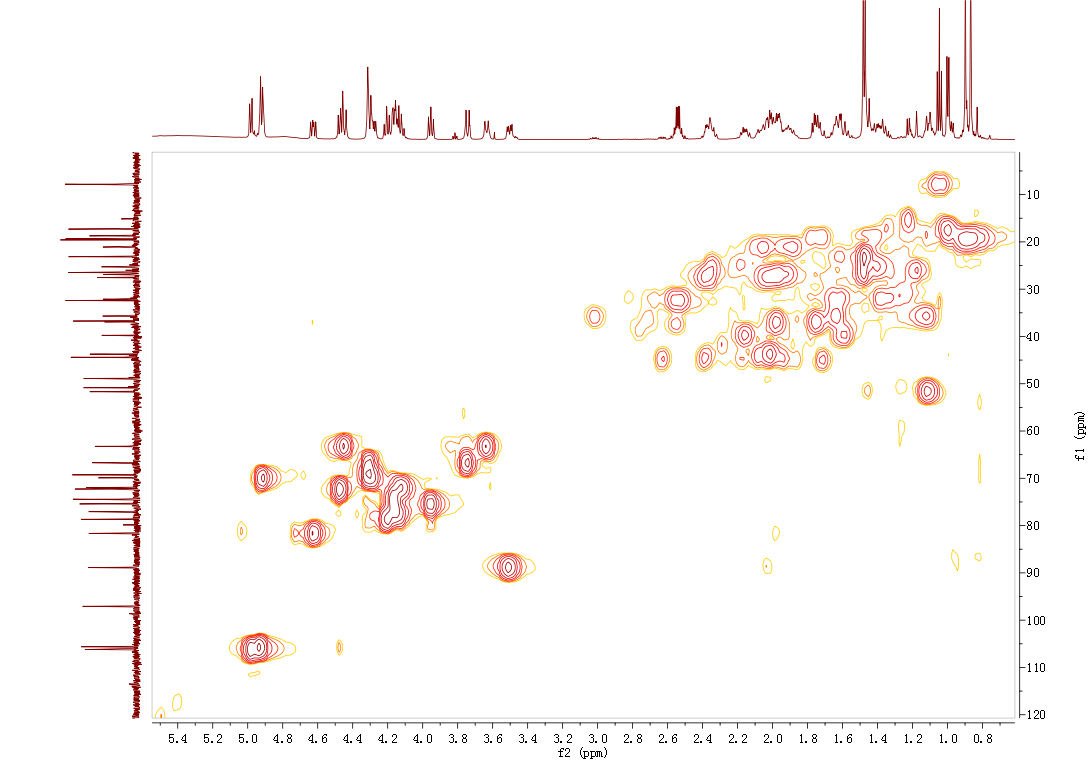

**S14.** HMBC spectrum (600 MHz, Pyridine-*d*_5_) of scillascilloside B-1 (**3**).


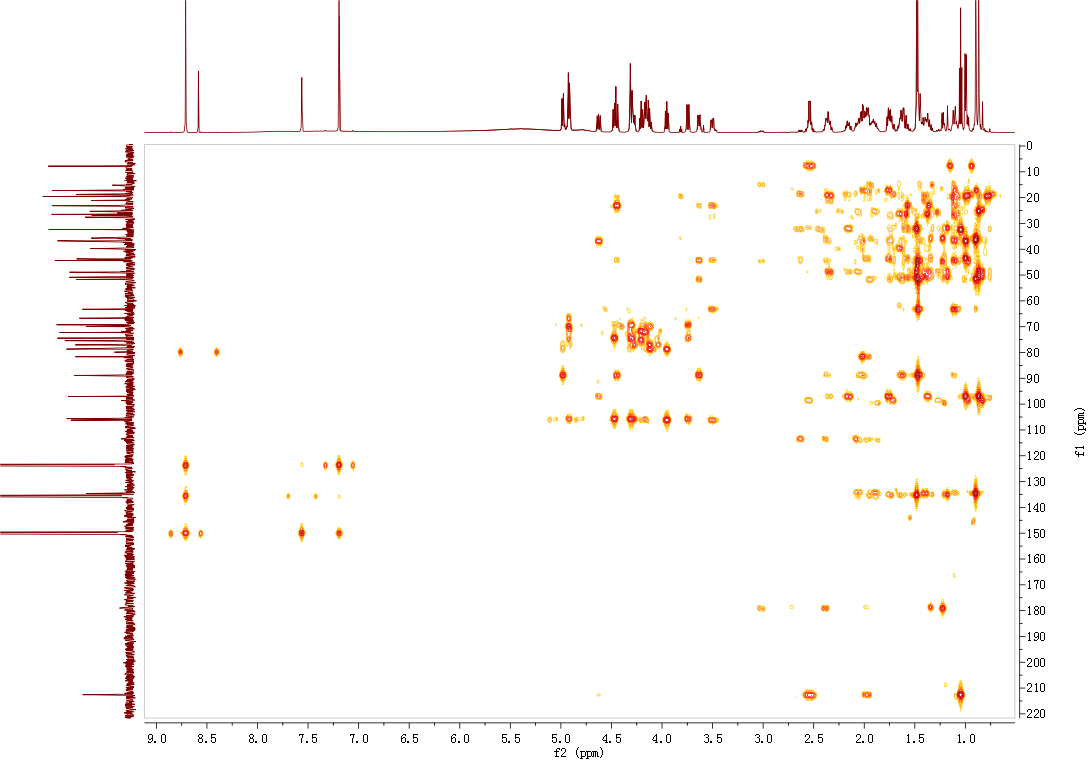

**S15.** ROESY spectrum (600 MHz, Pyridine-*d*_5_) of scillascilloside B-1 (**3**).


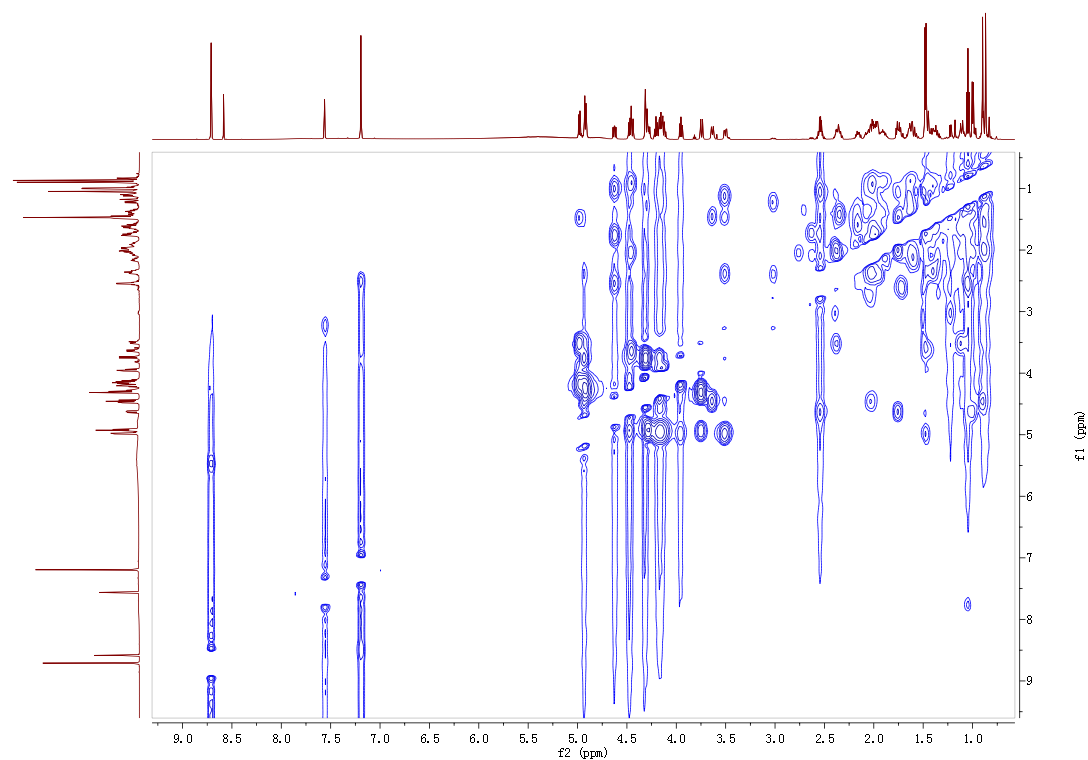

**S16.** ^1^H NMR spectrum (400 MHz, DMSO-*d*_6_) of drimiopsin D (**6**).


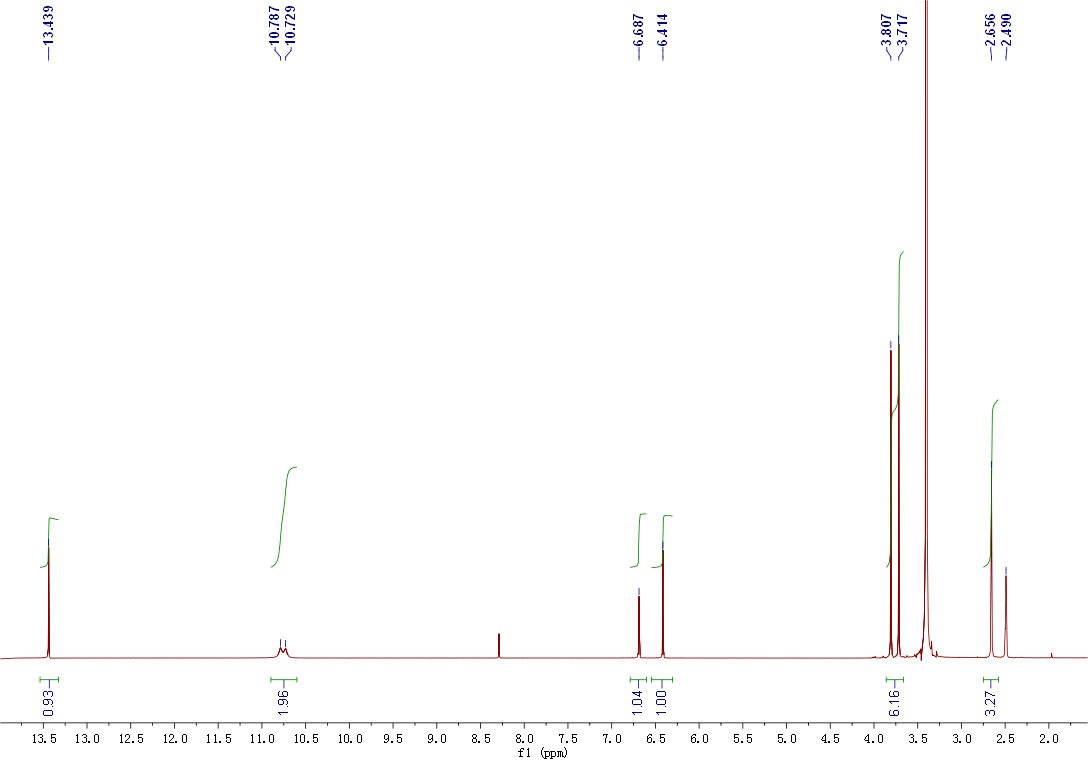

**S17.** ^13^C NMR spectrum (100 MHz, DMSO-*d*_6_) of drimiopsin D (**6**).


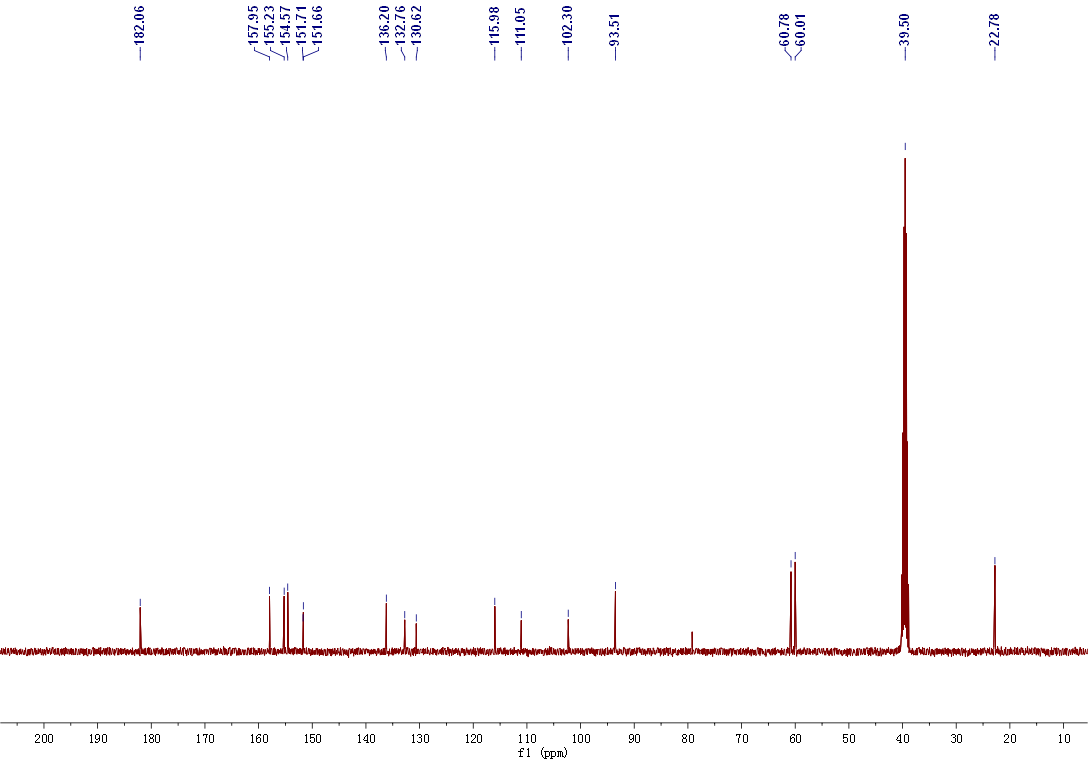

**S18.** HMBC spectrum (600 MHz, DMSO-*d*_6_) of drimiopsin D (**6**).


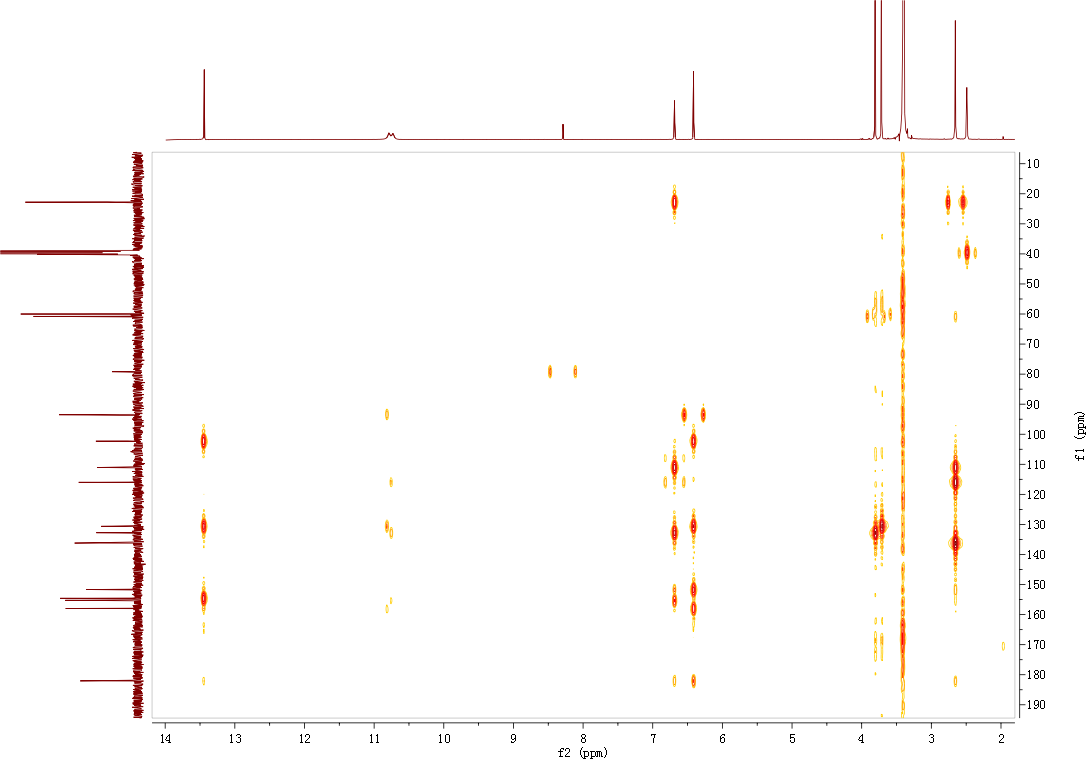

**S19.** ^1^H NMR spectrum (600 MHz, CD_3_OD) of drimiopsin D (**6**).


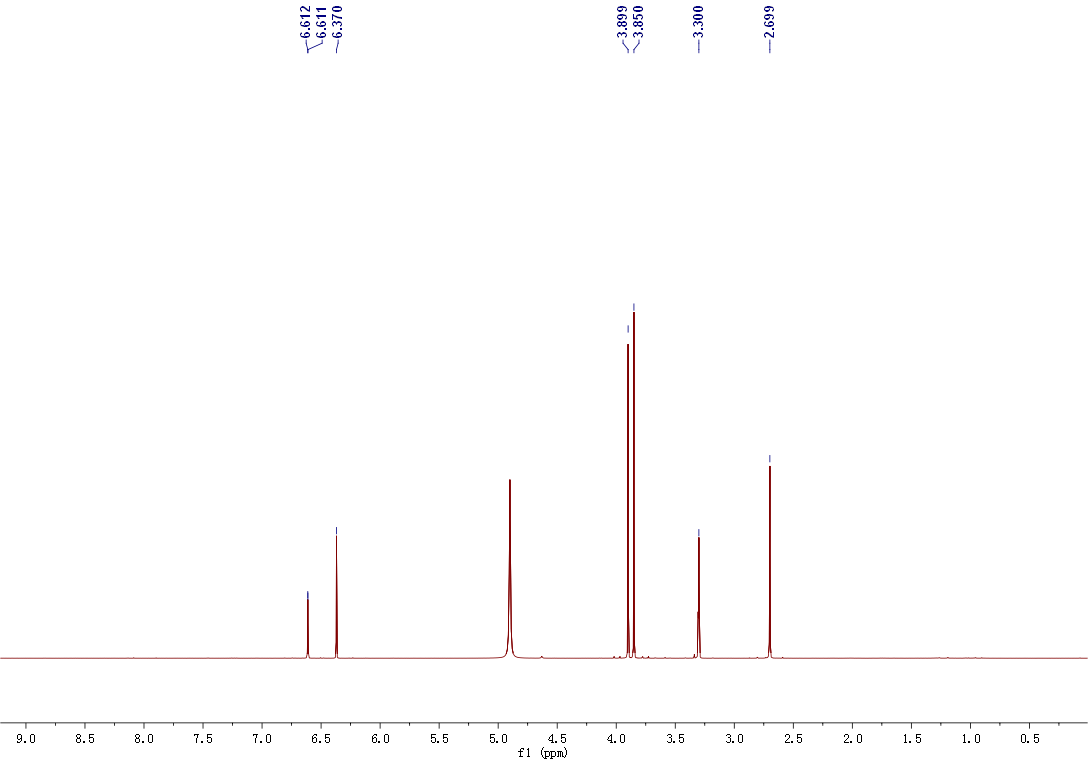

**S20.** ^13^C NMR spectrum (100 MHz, CD_3_OD) of drimiopsin D (**6**).


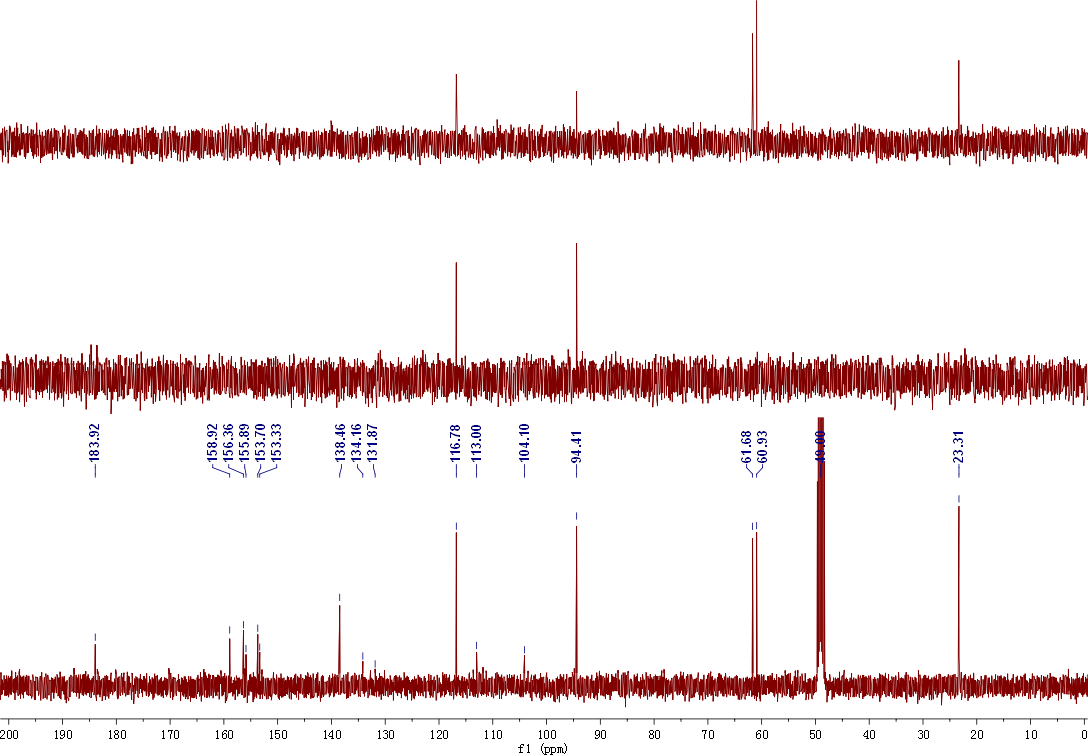

**S21.** HMBC spectrum (600 MHz, CD_3_OD) of drimiopsin D (**6**).


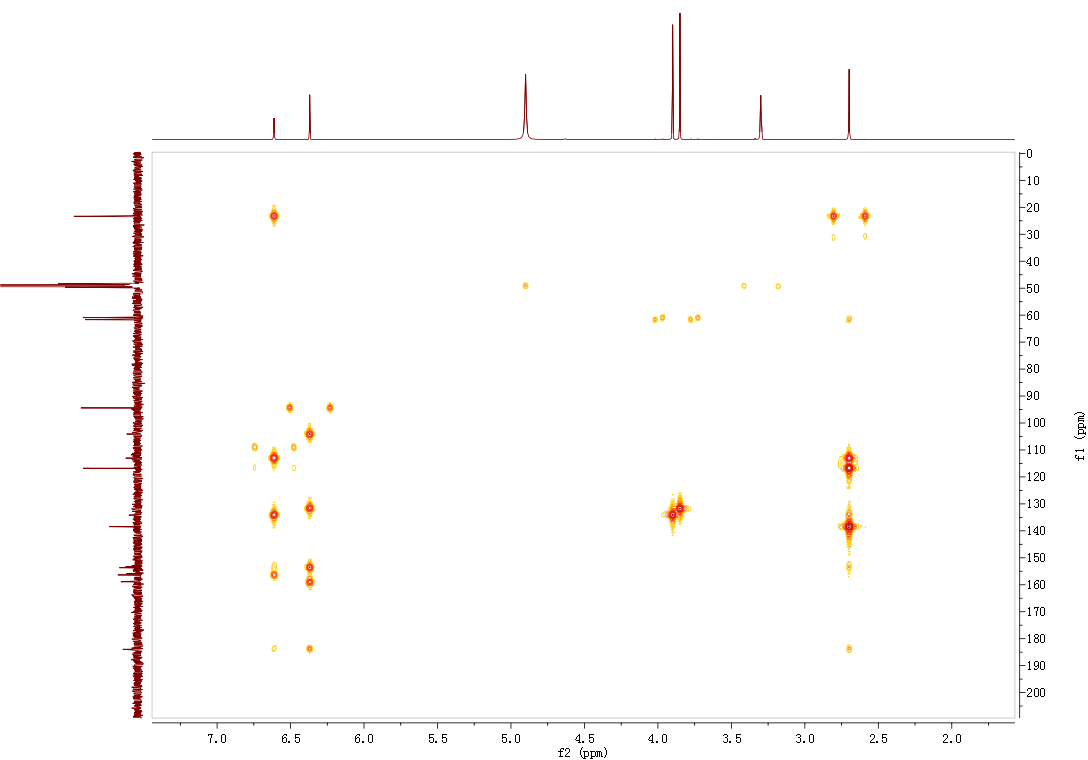

Supplement: Supplementary file 1 — Supplementary material 1 (DOCX 2005 kb) [file 13659_2015_76_MOESM1_ESM.docx]
